# Superresolution US Imaging of Rabbit Lymph Node Microvasculature for Metastasis Diagnosis

**DOI:** 10.1148/rycan.250674

**Published:** 2026-09-25

**Authors:** Hongsheng Ye, Xiaoxian Li, Chuan Peng, Qing Li, Liang Yang, Jichong He, Li Meng, Peng Li, Yixin Hu, Lingling Li, Jianhua Zhou

**Affiliations:** ^1^Department of Ultrasound, State Key Laboratory of Oncology in South China, Guangdong Provincial Clinical Research Center for Cancer, Sun Yat-sen University Cancer Center, No. 651 Dongfeng Road East, Guangzhou 510060, P. R. China; ^2^Department of Pathology, State Key Laboratory of Oncology in South China, Guangdong Provincial Clinical Research Center for Cancer, Sun Yat-sen University Cancer Center, Guangzhou, P. R. China

**Keywords:** Ultrasound-Contrast, Ultrasound

## Abstract

**Purpose:**

To compare the diagnostic performance of superresolution US (SRUS) with that of US and contrast-enhanced US (CEUS) for detecting lymph node (LN) metastasis in rabbits.

**Materials and Methods:**

US, CEUS, and SRUS were performed in 30 rabbits with 16 egg yolk–induced hyperplastic and 14 VX2 tumor–bearing metastatic LNs. Qualitative US, CEUS, and SRUS features and quantitative SRUS-based parameters of LN microvasculature were analyzed. Major qualitative features and quantitative parameters associated with metastatic LNs were identified, with histopathologic evaluation as reference. Spearman correlation between features and parameters was analyzed.

**Results:**

Among qualitative features, chaotic microvasculature at SRUS achieved the highest area under the receiver operating characteristic curve of 0.92 (95% CI: 0.81, 1.00), outperforming cortical thickening at US (0.67 [95% CI: 0.51, 0.82]; *P* = .012), arterial phase enhancing pattern at CEUS (0.72 [95% CI: 0.57, 0.86]; *P* = .018), and SRUS vascular pattern (0.72 [95% CI: 0.55, 0.89]; *P* = .028). Among quantitative parameters, fractal dimension yielded the optimal area under the receiver operating characteristic curve of 0.92 (95% CI: 0.79, 1.00), outperforming vessel density (0.70 [95% CI: 0.48, 0.88]; *P* = .046), flow-weighted vessel density (0.70 [95% CI: 0.49, 0.88]; *P* = .046), and direction entropy (0.71 [95% CI: 0.51, 0.89]; *P* = .046). Chaotic microvasculature and fractal dimension were strongly correlated (ρ = 0.80; *P* < .001), and both correlated well with microvascular density (ρ = 0.82 and 0.65, respectively; both *P* < .001).

**Conclusion:**

Chaotic microvasculature and fractal dimension assessed at SRUS showed improved diagnostic performance for detecting metastatic LNs in rabbits compared with US and CEUS and enabled noninvasive evaluation of microvascular tortuosity.

**Keywords:** Ultrasound-Contrast, Ultrasound

*Supplemental material is available for this article.*

© The Author(s) 2026. Published by the Radiological Society of North America under a CC BY 4.0 license.

## Graphical abstract

**Figure:**
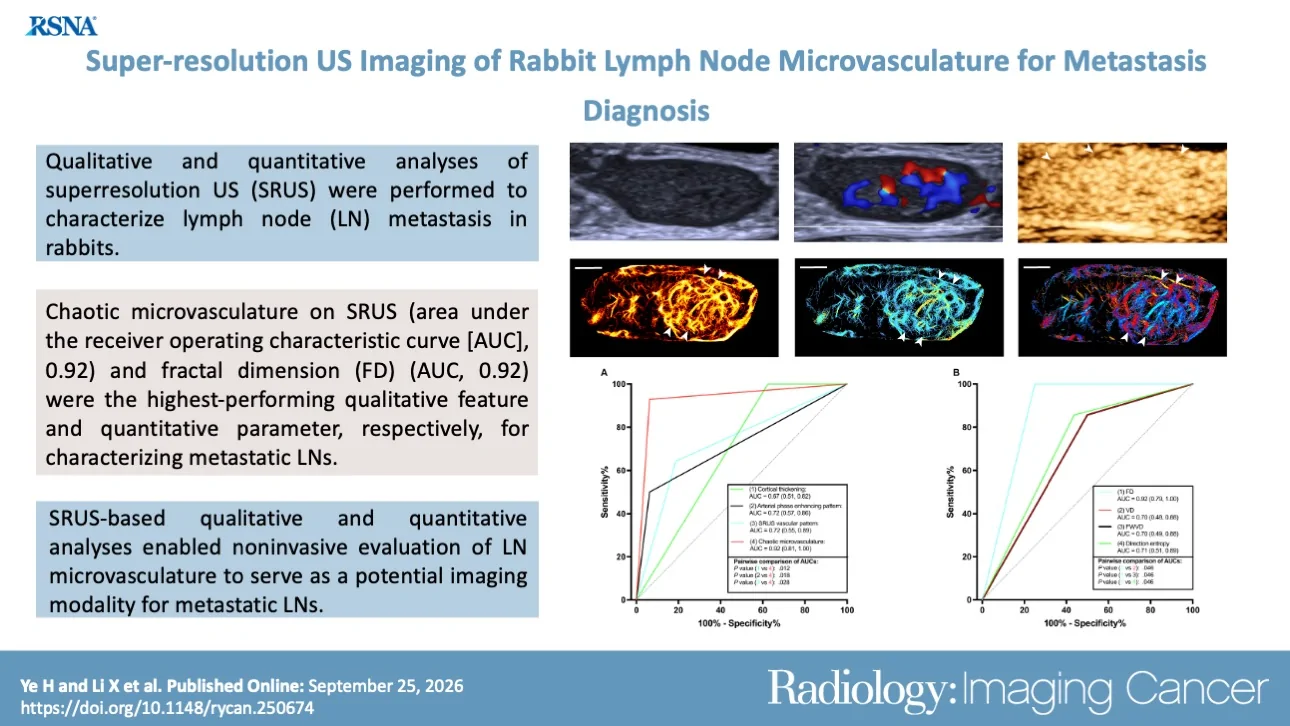

**Summary**

Qualitative and quantitative analyses showed that superresolution US enabled noninvasive evaluation of lymph node microvasculature in rabbits and that it can serve as a potential diagnostic modality for metastatic lymph nodes.

**Key Points**

■ In this in vivo imaging study of 30 rabbits with palpable lymph nodes, qualitative and quantitative analyses of superresolution US (SRUS) showed that chaotic microvasculature (area under the receiver operating characteristic curve, 0.92) and fractal dimension (area under the receiver operating characteristic curve, 0.92) were the highest-performing qualitative feature and quantitative parameter, respectively, for diagnosing metastatic lymph nodes.
■ Chaotic microvasculature at SRUS showed a strong positive correlation with fractal dimension (Spearman ρ = 0.80; *P* < .001), with both reflecting vessel tortuosity.
■ Both chaotic microvasculature and fractal dimension showed good correlations with microvascular density (ρ = 0.82 and 0.65, respectively; both *P* < .001).

## Introduction

Lymph node (LN) metastases in patients with cancer are associated with advanced tumor stage and poorer prognosis; the benefit of preoperative identification of LN metastases by imaging is that it allows clinicians to decide on appropriate treatment (1,2). Imaging findings on primary modalities, including US and dynamic contrast-enhanced MRI, that suggest metastatic LNs are marked enlargement and gross abnormalities in morphologic features, such as round shape and cortical thickening. However, most findings are nonspecific, which can be associated with hyperplastic LNs (3).

Primary tumors actively prime regional LNs by inducing a proangiogenic premetastatic niche before metastasis (4,5). Upon metastatic colonization, intranodal tumor growth triggers a robust angiogenic response, wherein rapid endothelial proliferation substantially outpaces the recruitment of supporting pericytes (6,7). Therefore, this tumor-induced neovasculature exhibits severe structural abnormalities, including high vessel density, tortuosity, and loss of normal hierarchy (8,9). Visualizing these specific microvascular abnormalities could aid in the characterization of metastatic LNs (10). Although some progress has been made in the clinical assessment of LN vasculature, limitations remain. Color Doppler flow imaging is sensitive to fast blood flow only in vessels larger than 0.2 mm in diameter (11,12). Contrast-enhanced US (CEUS) makes it possible to assess the tissue and tumor perfusion by means of real-time perfusion imaging (13). The effectiveness of the enhancement pattern at CEUS for the noninvasive assessment of LN metastasis has been demonstrated (14,15). However, CEUS is restricted to visualizing LN microvasculature abnormalities by its relatively low spatial resolution at submillimeter scale owing to the acoustic diffraction limit (16). Furthermore, CEUS primarily provides semiquantitative parameters, constraining the accurate quantification of microvascular hemodynamics (17,18). These limitations highlight a clinical need for a high-spatial-resolution imaging technique for the noninvasive evaluation of LN microvasculature.

Superresolution US (SRUS), also known as US localization microscopy, breaks through the acoustic diffraction limit by precisely locating and tracking intravascular microbubbles, thereby enabling in vivo high-resolution microvascular imaging at the micron scale (16,19–21). By accumulating microbubble localization tracks over time, SRUS generates high-resolution maps of microvascular intensity, velocity, and direction, providing a powerful tool for quantifying microvascular hemodynamics (22). Recent pilot studies have explored the initial feasibility of SRUS for visualizing hyperplastic and metastatic LN microvasculature (23,24). However, different quantitative parameters are defined across studies, with substantial value variations, which may limit the characterization of LN status. Another issue is that in previous studies, core biopsy was used as the reference standard, making it difficult to establish a clear correspondence between an area of microvascular abnormality and a metastatic region within an individual LN (25–27). Furthermore, based on SRUS microvasculature maps, visual assessment (qualitative analysis) of nodal microvascular abnormalities, such as loss of hierarchical branching and tortuous microvessels, could directly indicate metastatic LN involvement. To our knowledge, however, previous studies have not confirmed this.

In this study, we hypothesized that qualitative and quantitative assessment of LN microvasculature using SRUS would help to differentiate LN metastasis from hyperplasia. The aim of our study was to compare the diagnostic performance of SRUS for detecting LN metastasis in rabbits with that of US and CEUS.

## Materials and Methods

### Animal Model

All experimental procedures in this prospective study involving animals followed the guidelines of the Animal Research: Reporting of In Vivo Experiments imaging protocol (28) and were approved by Institutional Animal Care and Use Committee of Sun Yat-Sen University (SYSU-IACUC-2024-000945). The flowchart illustrating the experimental design is shown in Figure 1.

**Figure 1:**
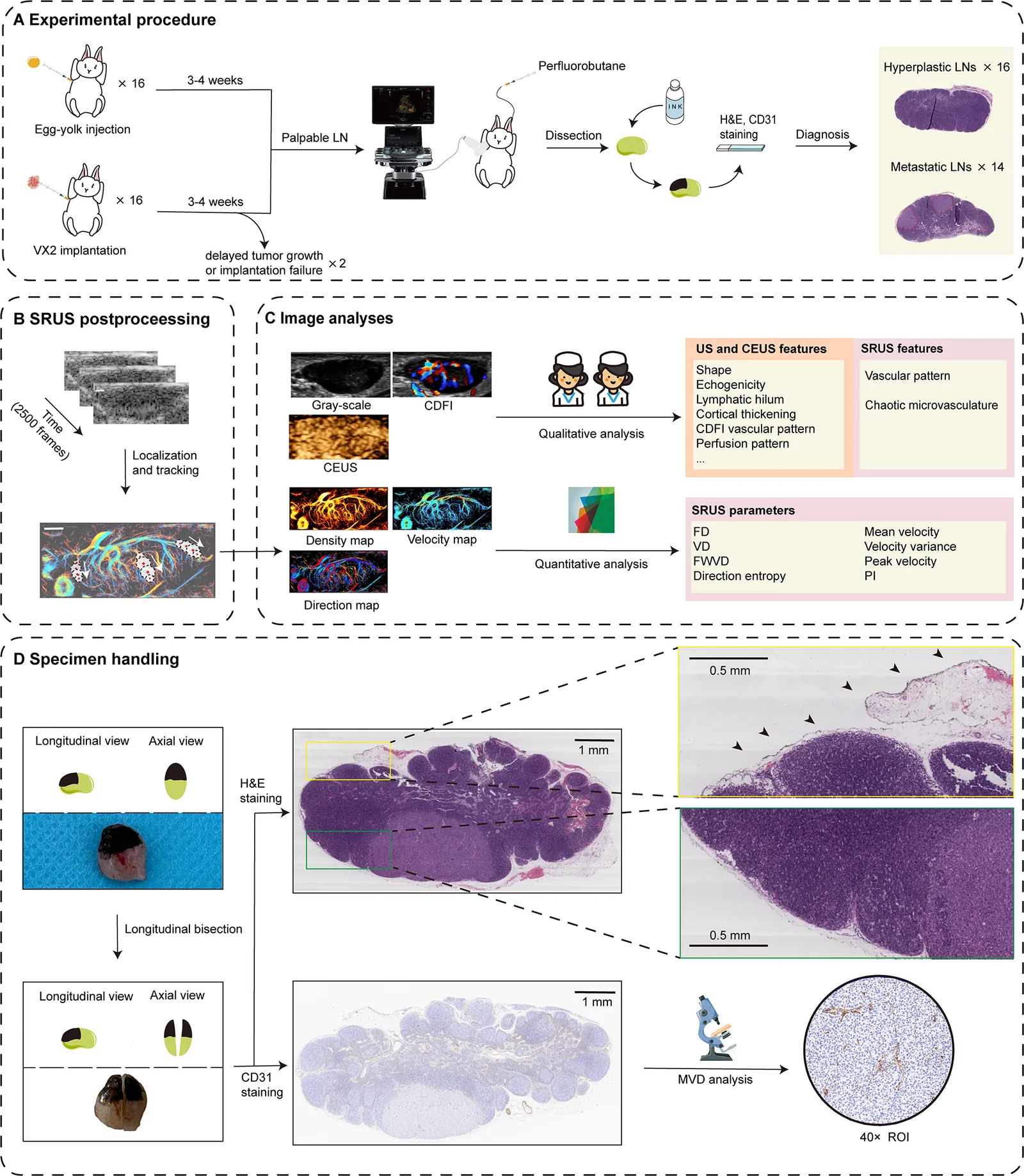
Diagram shows the experimental procedure for establishment of rabbit models of hyperplastic and metastatic lymph nodes (LNs) and LN image analyses. **(A)** Experimental procedure for establishment of rabbit models. **(B)** Superresolution US (SRUS) postprocessing procedure for LNs. **(C)** Image analysis procedure. Qualitative analysis was based on the US, contrast-enhanced US (CEUS), and SRUS features recorded by two expert radiologists. Quantitative analysis was based on SRUS parameters derived from SRUS maps. **(D)** Specimen handling procedure. Hematoxylin-eosin (H&E) and CD31 staining were performed. The yellow and green boxes represent enlarged regions of an H&E-stained section. The border of the LN in the yellow box was marked by ink (black arrowheads), and the border of the LN in the green box was not; this indicates that the histology section corresponds to the imaging plane (longitudinal view) of the LN. Microvascular density (MVD) analysis was performed within five hot spots at high magnification (×40). CDFI = color Doppler flow imaging, FD = fractal dimension, FWVD = flow-weighted vessel density, PI = perfusion index, ROI = region of interest, VD = vessel density. Scale bars = 1 mm.

Female New Zealand white rabbits (age, 3.5–4 weeks; weight, 2.5–3 kg; *n* = 32) were purchased from Guangdong Medical Laboratory Animal Center. Animals were housed at a constant room temperature (20–26°C) and humidity (40%–70%) with a 10-hour light, 14-hour dark cycle and were fed a standard diet. They were randomly assigned to a hyperplastic LN group (*n* = 16) or a metastatic LN group (*n* = 16). For hyperplastic LN modeling, each rabbit received subcutaneous 1.0-mL injections of 10% egg yolk into the right second mammary gland (29,30). Metastatic LN models were established by administering subcutaneous 1.0-mL injections of VX2 tumor suspension (JNO-Q0030-1; Jennio) into the right second mammary gland (31–34). Rabbits without palpable solid masses at the injection site 2 weeks after implantation were excluded because of delayed tumor growth or implantation failure. LNs were palpable 3–4 weeks after modeling and prepared for imaging. See Appendix S1 for detailed procedures of model establishment.

### Imaging Protocol

Each rabbit was imaged under anesthesia (maintained with 0.1 mL/kg/hr Zoletil50 solution; Virbac) (Appendix S1) using a clinical scanner (Resona A20; Mindray) equipped with an LM18-5WU linear array transducer for US and an SL10-3U linear array transducer for CEUS and SRUS; the latter lower-frequency transducer was used to better match the resonance frequency of perfluorobutane microbubbles (Sonazoid; GE HealthCare) and enhance signal detection (35). US was initially performed to localize the target palpable LN and determine the maximal cross-section, and US and color Doppler flow images were recorded. CEUS was performed using perfluorobutane microbubbles (0.04 mL/kg), and a continuous cine loop was acquired for approximately 60 seconds after injection (frame rate, 12 Hz; mechanical index, 0.129). After the initial 60 seconds, the SRUS protocol was immediately executed to acquire 5 seconds of raw data (frame rate, 500 Hz; mechanical index, 0.113). SRUS images, including density, velocity, and direction maps, were derived from SRUS raw data with SRCEUS Platform software (version 3.0; Mindray) (Fig 1B). Protocol details are presented in Appendix S2.

### Qualitative Analysis

On US images, five qualitative features of each LN were reviewed (36,37): *(a) *shape (oval or round), *(b) *echogenicity (homogeneous or heterogeneous), *(c)* lymphatic hilum (present or absent), *(d) *cortical thickening (no thickening, eccentric thickening, or diffuse uniform thickening), and *(e) *color Doppler flow imaging vascular pattern (hilar, peripheral, mixed, or indeterminate). On CEUS images, three qualitative features of LNs were reviewed (38): *(a) *perfusion pattern (centrifugal, integral, centripetal, or asynchronous), *(b) *arterial phase enhancing pattern (homogeneous or heterogeneous), and *(c) *perfusion defect (absent or present). On SRUS images, two SRUS features were reviewed (8,37,39): *(a) *SRUS vascular pattern (hilar, peripheral, mixed, or indeterminate) and *(b) *chaotic microvasculature (absent, focal, or integral). Hilar vascular pattern was characterized by one or a few vessels entering through the LN hilum and branching radially into the parenchyma in a centrifugal direction, whereas peripheral vascular pattern was identified by vessels originating from the periphery of the LN and extending toward the hilum in a centripetal direction. If both were present, a mixed vascular pattern was established (Fig 2A). LNs exhibiting microvessel distortion with loss of hierarchical branching on SRUS images were defined as having chaotic microvasculature. If present, this pattern was further categorized as focal when distortion affected only a portion of the microvasculature or integral when observed throughout the entire microvessel network (Fig 2B).

**Figure 2:**
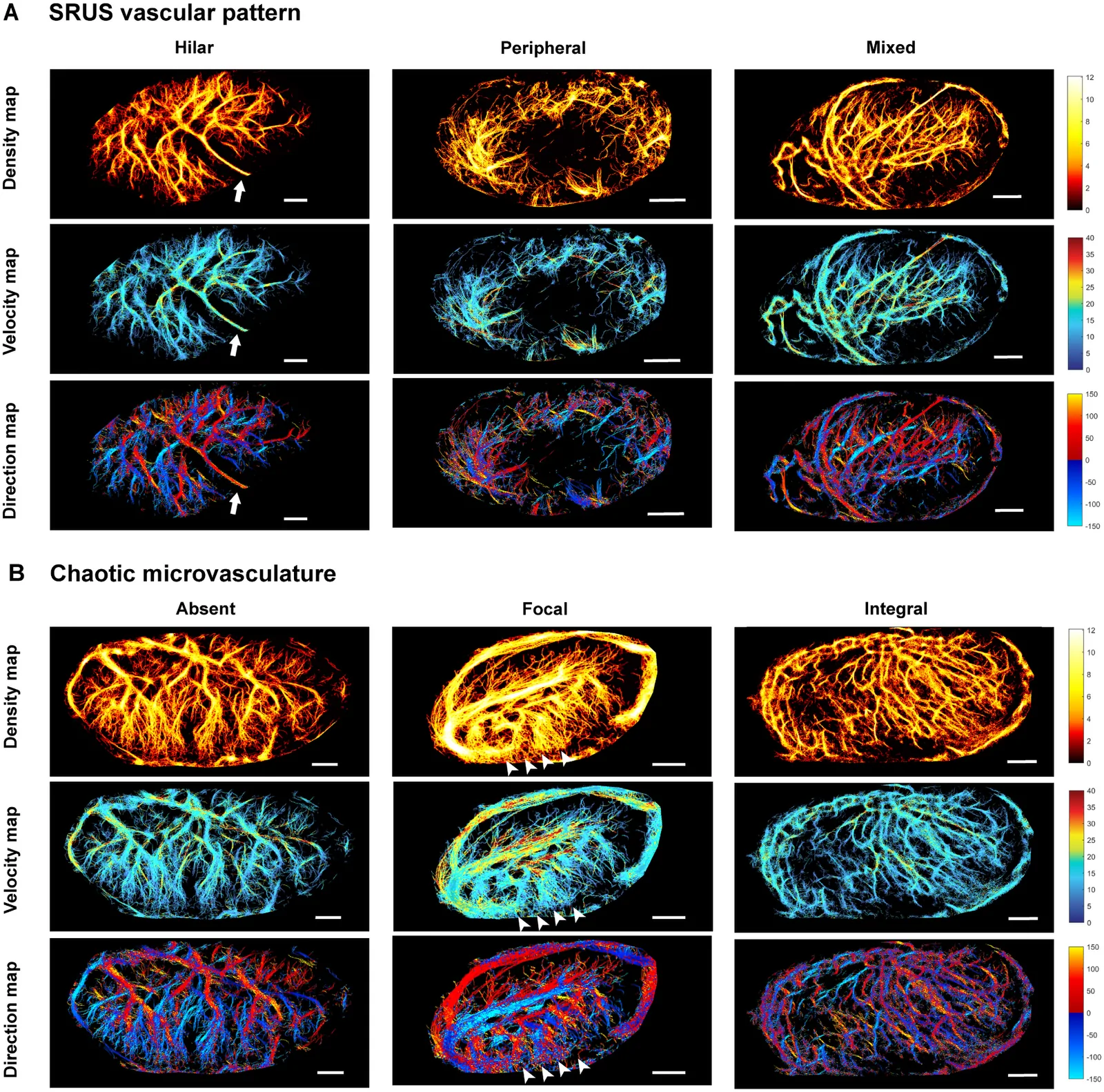
Superresolution US (SRUS) images including density, velocity, and direction maps. **(A)** SRUS vascular pattern of lymph nodes (LNs), recorded as hilar (left panels, arrows indicate the LN hilum), peripheral (middle panels), mixed vascular pattern (right panels), or failure to determine (not presented). **(B)** Manifestations of chaotic microvasculature in LNs, recorded as absent (left panels), focal (middle panels, arrowheads), or integral (right panels). Scale bars = 1 mm.

Two expert radiologists (Q.L. and C.P., with 13 and 12 years of experience in US, respectively) blinded to group assignment independently reviewed all US, CEUS and SRUS images and assessed qualitative features (Fig 1C). Disagreements were resolved by consultation with a third expert radiologist (J.Z., with 24 years of experience in US). The major qualitative features associated with metastatic LNs were identified, as described later in the Statistical Analysis section.

### Quantitative Analysis

A radiologist (Y.H., with 6 years of experience in US), also blinded to group assignment, manually delineated the regions of interest of each LN on SRUS maps three times. These regions of interest were then analyzed by SRCEUS Platform software to generate SRUS quantitative parameters. For each LN, quantitative parameters derived from triplicate region-of-interest delineations were averaged for subsequent analyses.

Eight SRUS quantitative parameters were analyzed (22): *(a) *fractal dimension (FD), defined as vascular morphologic complexity calculated by a box-counting algorithm; *(b) *vessel density (VD), expressed as the ratio of blood vessel count to the LN total area; *(c) *flow-weighted vessel density (FWVD), representing perfused blood volume; *(d) *direction entropy, quantified as information entropy of flow direction distribution; *(e) *mean velocity; *(f) *peak velocity; *(g) *velocity variance, measuring variance of flow velocities; and *(h) *perfusion index, calculated as VD × mean velocity to reflect microcirculatory perfusion. See Appendix S3 for detailed definitions and calculations of these quantitative parameters. The relevant SRUS quantitative parameters associated with metastatic LNs were identified, as described later in the Statistical Analysis section.

### Reference Standard

After imaging, rabbits were euthanized by intravenous overdose of anesthesia (0.8 mL/kg Zoletil50 solution). LNs were then resected under US guidance. The dissected LNs were marked with histocompatible ink to visually indicate the orientation of imaging; this orientation served as a reference for sectioning alignment with the maximal cross-section of imaging plane during tissue processing (Fig 1D). Dissected LNs were then fixed in formalin, embedded in paraffin, and sliced along the ink marking. A set of five consecutive slices were acquired per node, with a slice thickness of 2–3 μm. Definitive pathology reference standards were established on the basis of hematoxylin-eosin–stained slices, reviewed by a pathologist (P.L., 10 years of experience in LN pathology) blinded to group assignment. Metastatic involvement was defined as the presence of macroscopic tumor deposits (>2 mm) according to the *American Joint Committee on Cancer 8th Edition Staging Manual* (40).

Immunostaining of vascular endothelial cells in LN was performed with CD31 antibody (1:200, bsm-4366M; Bioss) as a marker of microvasculature reference for calculating microvascular density (MVD), followed by incubation with a horseradish peroxidase–conjugated secondary antibody (1:2000, ab205718; Abcam). MVD was assessed using CaseViewer software (version 2.4; 3DHistech) in accordance with the Weidner method (41). For each LN section, five regions of high MVD (hot spots) were initially identified by the same pathologist (P.L.) at low magnification (×20). Subsequently, vessel counts were performed within each hot spot at high magnification (×40), and vessel counts derived from five hot spots were averaged as the MVD value (Fig 1D).

### Statistical Analysis

Sample size was calculated using software (PASS 2023; NCSS). Categorical variables are presented as frequencies with percentages. Continuous variables are reported as means ± SDs or medians with IQRs, as appropriate, and were compared between groups using the Student *t* test or Mann-Whitney *U* test. Interreader agreement of individual qualitative features between the two independent radiologists was assessed using κ statistics with 95% CIs, and intrareader reproducibility of three measurements of quantitative parameters was calculated using intraclass correlation coefficients (ICCs) with 95% CIs. κ coefficients were categorized according to Landis and Koch using the following criteria (42): 0.00–0.20, slight agreement; 0.21–0.40, fair agreement; 0.41–0.60, moderate agreement; 0.61–0.80, good agreement; and 0.81–1.00, excellent agreement. ICCs were interpreted according to Koo and Li as follows (43): less than 0.50, poor; 0.50–0.75, moderate; 0.76–0.90, good; and greater than 0.90, excellent. Major qualitative features and relevant SRUS quantitative parameters were identified using univariable binary logistic regression analysis, with the odds ratios (ORs) corrected using the Firth method and internally validated using 1000 bootstrap samples. The diagnostic performance of major qualitative features and relevant SRUS quantitative parameters for differentiating metastatic from benign LNs was evaluated, with the area under the receiver operating characteristic curve (AUC) serving as the overall performance measure. The optimal cutoff values of relevant SRUS quantitative parameters were determined according to the Youden index. The 95% CIs for sensitivity, specificity, positive predictive value, negative predictive value, and AUCs were computed and compared using the bootstrap method with 1000 resamples. Correlations between major qualitative features and relevant SRUS quantitative parameters, and correlations of major features and relevant parameters with MVD were analyzed with the Spearman correlation coefficient (ρ) according to Schober as follows (44): 0.00–0.09, negligible; 0.10–0.39, weak; 0.40–0.69, moderate; 0.70–0.89, strong; and 0.90–1.00, very strong correlation. *P* values were adjusted with the false discovery rate method for multiple comparisons. A two-tailed *P* value less than .05 was considered to indicate a statistically significant difference. All statistical analyses were performed with SPSS software (version 31.0; IBM), and Python (version 3.12.7; Python Software Foundation) by two authors (H.Y. and X.L.). For more details about the statistical analysis plan, see Appendix S4.

## Results

### Animals and Target LNs

In this study, a total of 30 rabbits with 30 palpable LNs (16 hyperplastic and 14 metastatic LNs; long axis, 1.05 cm [IQR, 0.94–1.26 cm]; short axis, 0.38 cm [IQR, 0.30–0.53 cm]) were included in the final analysis after the exclusion of two rabbits with tumor implantation failure. Hyperplastic LN sizes were similar to those of metastatic LNs (long axis, 1.02 cm [IQR, 0.94–1.11 cm] vs 1.17 cm [IQR, 0.94–1.29 cm]; *P* = .43; short axis, 0.36 cm [IQR, 0.30–0.48 cm] vs 0.41 cm [IQR, 0.33–0.58 cm]; *P* = .29).

### Major Qualitative Features Associated with Metastatic LNs

As shown in Table 1, four qualitative features were identified as major features associated with metastatic LNs in the univariable analysis, including cortical thickening at US (OR, 17.95 [95% CI: 5.56, 57.00]; *P* = .002), arterial phase enhancing pattern at CEUS (OR, 10.33 [95% CI: 2.44, 77.00]; *P* = .001), SRUS vascular pattern (OR, 6.66 [95% CI: 1.68, 52.20]; *P* = .004), and chaotic microvasculature at SRUS (OR, 93.00 [95% CI: 18.82, 957.00]; *P* = .001).

**Table 1:** Univariable Analysis of US, CEUS, and SRUS Features of Rabbit Lymph Nodes

Feature	Hyperplasia (*n* = 16)	Metastasis (*n* = 14)	Univariable Analysis	
			Odds Ratio (95% CI)*	*P* Value*
US				
L (cm^)^^†^	1.02 (0.94–1.11)	1.17 (0.94–1.29)	1.26 (0.64, 4.80)	.43
S (cm)^†^	0.36 (0.30–0.48)	0.41 (0.33–0.58)	1.74 (0.83, 3.68)	.29
L:S ratio^†^	2.67 (2.13–3.68)	2.39 (2.07–3.07)	0.69 (0.27, 1.48)	.38
Shape			0.77 (0.10, 6.52)	.85
Oval^‡^	13 (81)	12 (86)		
Round	3 (19)	2 (14)		
Echogenicity			6.60 (1.00, 21.25)	.26
Homogeneous^‡^	16 (100)	12 (86)		
Heterogeneous	0 (0)	2 (14)		
Lymphatic hilum			2.96 (0.77, 18.46)	.13
Present^‡^	9 (56)	4 (29)		
Absent	7 (44)	10 (71)		
Cortical thickening			17.95 (5.56, 57.00)	.002^§^
No thickening^‡^	6 (38)	0 (0)		
Eccentric thickening	1 (6)	2 (14)		
Diffuse uniform thickening	9 (56)	12 (86)		
CDFI vascular pattern			4.14 (0.91, 36.74)	.06
Hilar^‡^	14 (88)	8 (58)		
Peripheral	0 (0)	2 (14)		
Mixed	0 (0)	2 (14)		
Indeterminate	2 (12)	2 (14)		
CEUS				
Perfusion pattern			1.77 (0.24, 13.63)	.59
Centrifugal^‡^	11 (69)	8 (57)		
Integral^‡^	3 (19)	3 (21)		
Centripetal	1 (6)	2 (14)		
Asynchronous	1 (6)	1 (7)		
Arterial phase enhancing pattern			10.33 (2.44, 77.00)	.001^§^
Homogeneous^‡^	15 (94)	7 (50)		
Heterogeneous	1 (6)	7 (50)		
Perfusion defect			2.07 (0.24, 16.06)	.51
Absent^‡^	15 (94)	12 (86)		
Present	1 (6)	2 (14)		
SRUS				
SRUS vascular pattern			6.66 (1.68, 52.20)	.004^§^
Hilar^‡^	13 (81)	5 (36)		
Peripheral	0 (0)	1 (7)		
Mixed	3 (19)	8 (57)		
Indeterminate	0 (0)	0 (0)		
Chaotic microvasculature			93.00 (18.82, 957.00)	.001^§^
Absent^‡^	15 (94)	1 (7)		
Focal	0 (0)	4 (29)		
Integral	1 (6)	9 (64)		

Note.—Unless otherwise indicated, data are medians with IQRs in parentheses or numbers of lymph nodes with percentages in parentheses. CDFI = color Doppler flow imaging, CEUS = contrast-enhanced US, L = long-axis diameters, S = short-axis diameters, SRUS = superresolution US.

* Obtained from univariable logistic regression models using Firth penalized likelihood using bootstrap based on 1000 resamples.

^†^ Compared with the Mann-Whitney *U* test.

^‡^ As reference.

^§^ Statistically significant.

### Interreader Agreement of Qualitative Features

Table S1 summarizes interreader agreement (κ values and agreement level categories) for qualitative features at US, CEUS, and SRUS. For the four major qualitative features, interreader agreement was fair for cortical thickening at US (κ = 0.40 [95% CI: 0.04, 0.71]) and moderate for arterial phase enhancing pattern at CEUS (κ = 0.55 [95% CI: 0.21, 0.84]), SRUS vascular pattern (κ = 0.51 [95% CI: 0.15, 0.83]), and chaotic microvasculature at SRUS (κ = 0.60 [95% CI: 0.27, 0.87]).

### Diagnostic Performance of Major Qualitative Features

Diagnostic performance metrics for the four major qualitative features for metastatic LN diagnosis are shown in Table 2. For cortical thickening at US, sensitivity was 100% (14 of 14; 95% CI: 78, 100), specificity was 38% (six of 16; 95% CI: 13, 62), and the AUC was 0.67 (95% CI: 0.51, 0.82); for arterial phase enhancing pattern at CEUS, these values were 50% (seven of 14; 95% CI: 22, 75), 94% (15 of 16; 95% CI: 79, 100), and 0.72 (95% CI: 0.57, 0.86); for SRUS vascular pattern, the values were 64% (nine of 14; 95% CI: 36, 91), 81% (13 of 16; 95% CI: 60, 100), and 0.72 (95% CI: 0.55, 0.89); and for chaotic microvasculature at SRUS, the values were 93% (13 of 14; 95% CI: 75, 100), 94% (15 of 16; 95% CI: 78, 100), and 0.92 (95% CI: 0.81, 1.00), respectively.

**Table 2:** Performance of Major Features for Diagnosis of Metastasis in Rabbit Lymph Nodes

Feature	Sensitivity (%)	Specificity (%)	PPV (%)	NPV (%)	AUC*
US					
1: Cortical thickening	100 (78, 100) [14/14]	38 (13, 62) [6/16]	58 (39, 78) [14/24]	100 (61, 100) [6/6]	0.67 (0.51, 0.82)
CEUS					
2: Arterial phase enhancing pattern	50 (22, 75) [7/14]	94 (79, 100) [15/16]	88 (57, 100) [7/8]	68 (48, 86) [15/22]	0.72 (0.57, 0.86)
SRUS					
3: SRUS vascular pattern	64 (36, 91) [9/14]	81 (60, 100) [13/16]	75 (50, 100) [9/12]	72 (50, 93) [13/18]	0.72 (0.55, 0.89)
4: Chaotic microvasculature	93 (75, 100) [13/14]	94 (78, 100) [15/16]	93 (77, 100) [13/14]	94 (79, 100) [15/16]	0.92 (0.81, 1.00)
*P* value^†^					
1 vs 4	.67	< .001^‡^	–	–	.012^‡^
2 vs 4	.006^‡^	> .99	–	–	.018^‡^
3 vs 4	.17	.39	–	–	.028^‡^

Note.—Unless otherwise indicated, data are percentages with 95% CIs in parentheses and numbers of lymph nodes in brackets. AUC = area under the receiver operating characteristic curve, CEUS = contrast-enhanced US, NPV = negative predictive value, PPV = positive predictive value, SRUS = superresolution US.

* AUCs were calculated using the Dorfman-Berbaum-Metz method with Hillis improvements.

^†^ *P* values were computed using 1000-resample bootstrap and adjusted for multiple comparisons using false discovery rate.

^‡^ Statistically significant.

Comparing the diagnostic performance of these four major qualitative features, chaotic microvasculature at SRUS achieved the optimal AUC, outperforming cortical thickening at US (0.92 vs 0.67; *P* = .012), arterial phase enhancing pattern at CEUS (0.92 vs 0.72; *P* = .018), and SRUS vascular pattern (0.92 vs 0.72; *P* = .028). The receiver operating characteristic curves for this comparison are shown in Figure S1A.

### Relevant SRUS Quantitative Parameters Associated with Metastatic LNs

As shown in Figure 3 (also see Table 3), four of the eight quantitative parameters derived from SRUS maps were significantly higher in metastatic LNs than in hyperplastic LNs. These parameters—FD, VD, FWVD, and direction entropy—were identified as relevant SRUS quantitative parameters associated with metastatic LNs (FD, hyperplasia vs metastasis, 1.67 ± 0.05 vs 1.76 ± 0.04; OR, 17.46 [95% CI: 5.85, 1872.62]; *P* = .001; VD, 0.50 ± 0.12 vs 0.58 ± 0.08; OR, 2.18 [95% CI: 1.13, 6.27]; *P* = .02; FWVD, 2.10 ± 0.65 vs 2.55 ± 0.42; OR, 2.27 [95% CI: 1.12, 6.53]; *P* = .02; direction entropy, 0.80 ± 0.11 vs 0.87 ± 0.07; OR, 2.32 [95% CI: 1.19, 8.09]; *P* = .03).

**Figure 3:**
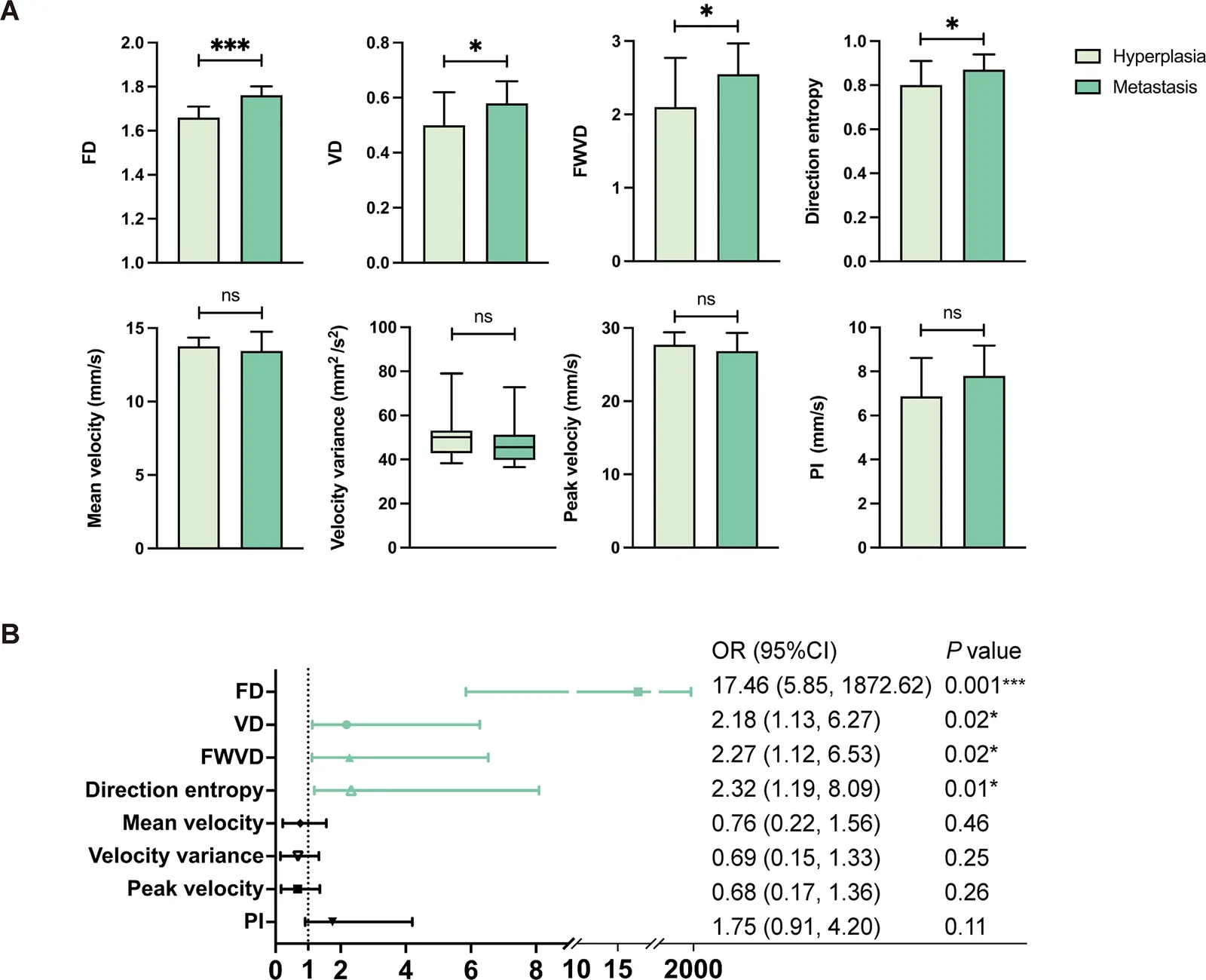
**(A)** Comparison of superresolution US (SRUS) quantitative parameters between hyperplastic and metastatic rabbit lymph nodes. Bars represent means, and error bars represent the SD. Boxes represent IQRs, middle lines represent medians, and whiskers indicate 1.5 × IQR. **(B)** Forest plot shows the results of the univariable analysis of SRUS quantitative parameters. *P < .05, **P < .01, ***P ≤ .001. FD = fractal dimension, FWVD = flow-weighted vessel density, OR = odds ratio, PI = perfusion index, VD = vessel density, ns = nonsignificant.

**Table 3:** Comparison of Superresolution US Quantitative Parameters between Hyperplastic and Metastatic Rabbit Lymph Nodes

Parameter	Hyperplasia (*n* = 16)	Metastasis (*n* = 14)	*P* Value
FD	1.67 ± 0.05	1.76 ± 0.04	<.001*
VD	0.50 ± 0.12	0.58 ± 0.08	.04*
FWVD	2.10 ± 0.65	2.55 ± 0.42	.03*
Direction entropy	0.80 ± 0.11	0.87 ± 0.07	.03*
Mean velocity (mm/sec)	13.74 ± 0.61	13.44 ± 1.31	.40
Velocity variance (mm^2^/sec^−2^)	50.15 (43.27–52.66)	45.51 (39.86–49.33)	.15^†^
Peak velocity (mm/sec)	27.70 ± 1.71	26.83 ± 2.50	.27
PI	6.87 ± 1.74	7.80 ± 1.38	.12

Note.—Data are reported as means ± SDs or medians with IQRs in parentheses and were compared between groups using the Student *t* test or Mann-Whitney *U* test. FD = fractal dimension, FWVD = flow-weighted vessel density, PI = perfusion index, VD = vessel density.

* Statistically significant.

^†^ Obtained from the Mann-Whitney *U* test.

### Intrareader Reproducibility of Quantitative Parameters

Table S2 summarizes the intrareader reproducibility (ICC values and reproducibility categories) for SRUS quantitative parameters. For the four relevant quantitative parameters, intrareader reproducibility was good for FD (ICC, 0.86 [95% CI: 0.76, 0.93]) and direction entropy (ICC, 0.88 [95% CI: 0.80, 0.94]) and excellent for VD (ICC, 0.92 [95% CI: 0.85, 0.96]) and FWVD (ICC, 0.92 [95% CI: 0.86, 0.96]).

### Diagnostic Performance of Relevant SRUS Quantitative Parameters

The diagnostic performance metrics of the four relevant SRUS quantitative parameters associated with metastatic LNs are shown in Table 4. For FD, sensitivity was 100% (14 of 14; 95% CI: 78, 100), specificity was 81% (13 of 16; 95% CI: 62, 100), and the AUC was 0.92 (95% CI: 0.79, 1.00); for VD, these values were 93% (13 of 14; 95% CI: 75, 100), 50% (eight of 16; 95% CI: 25, 75), and 0.70 (95% CI: 0.48, 0.88); for FWVD, the values were 93% (13 of 14; 95% CI: 75, 100), 56% (nine of 16; 95% CI: 31, 81), and 0.70 (95% CI: 0.49, 0.88); and for direction entropy, the values were 93% (13 of 14; 95% CI: 75, 100), 50% (eight of 16; 95% CI: 25, 75), and 0.71 (95% CI: 0.51, 0.89).

**Table 4:** Performance of Relevant SRUS Parameters for Diagnosis of Metastasis in Rabbit Lymph Nodes

SRUS Parameter	Sensitivity (%)	Specificity (%)	PPV (%)	NPV (%)	AUC
1: FD	100 (78, 100) [14/14]	81 (62, 100) [13/16]	82 (62, 100) [14/17]	100 (77, 100) [13/13]	0.92 (0.79, 1.00)
2: VD	93 (75, 100) [13/14]	50 (25, 75) [8/16]	62 (39, 81) [13/21]	89 (62, 100) [8/9]	0.70 (0.48, 0.88)
3: FWVD	93 (75, 100) [13/14]	56 (31, 81) [9/16]	65 (41, 85) [13/20]	90 (67, 100) [9/10]	0.70 (0.49, 0.88)
4: Direction entropy	93 (75, 100) [13/14]	50 (25, 75) [8/16]	62 (39, 81) [13/21]	89 (62, 100) [8/9]	0.71 (0.51, 0.89)
*P* value*					
1 vs 2	.716	.069	…	…	.046^†^
1 vs 3	.716	.118	…	…	.046^†^
1 vs 4	.716	.069	…	…	.046^†^

Note.—Data are percentages with 95% CIs in parentheses and numbers of lymph nodes in brackets. AUC = area under the receiver operating characteristic curve, FD = fractal dimension, FWVD = flow-weighted vessel density, NPV = negative predictive value, PPV = positive predictive value, SRUS = superresolution US, VD = vessel density.

* *P* values were computed using 1000-resample bootstrap and adjusted for multiple comparisons using false discovery rate.

^†^ Statistically significant.

Comparing the diagnostic performance of these SRUS quantitative parameters, FD achieved the optimal AUC, outperforming VD (0.92 vs 0.70; *P* = .046), FWVD (0.92 vs 0.70; *P* = .046), and direction entropy (0.92 vs 0.71; *P* = .046). The receiver operating characteristic curves for this comparison are shown in Figure S1B.

### Correlation between Major Qualitative Features and Relevant SRUS Quantitative Parameters

The matrix for the correlation between the four major qualitative features and the four relevant SRUS quantitative parameters is shown in Figure 4. Chaotic microvasculature at SRUS and FD—the highest-performing qualitative features and SRUS quantitative parameters, respectively—showed a strong positive correlation (Spearman ρ = 0.80; *P* < .001).

**Figure 4:**
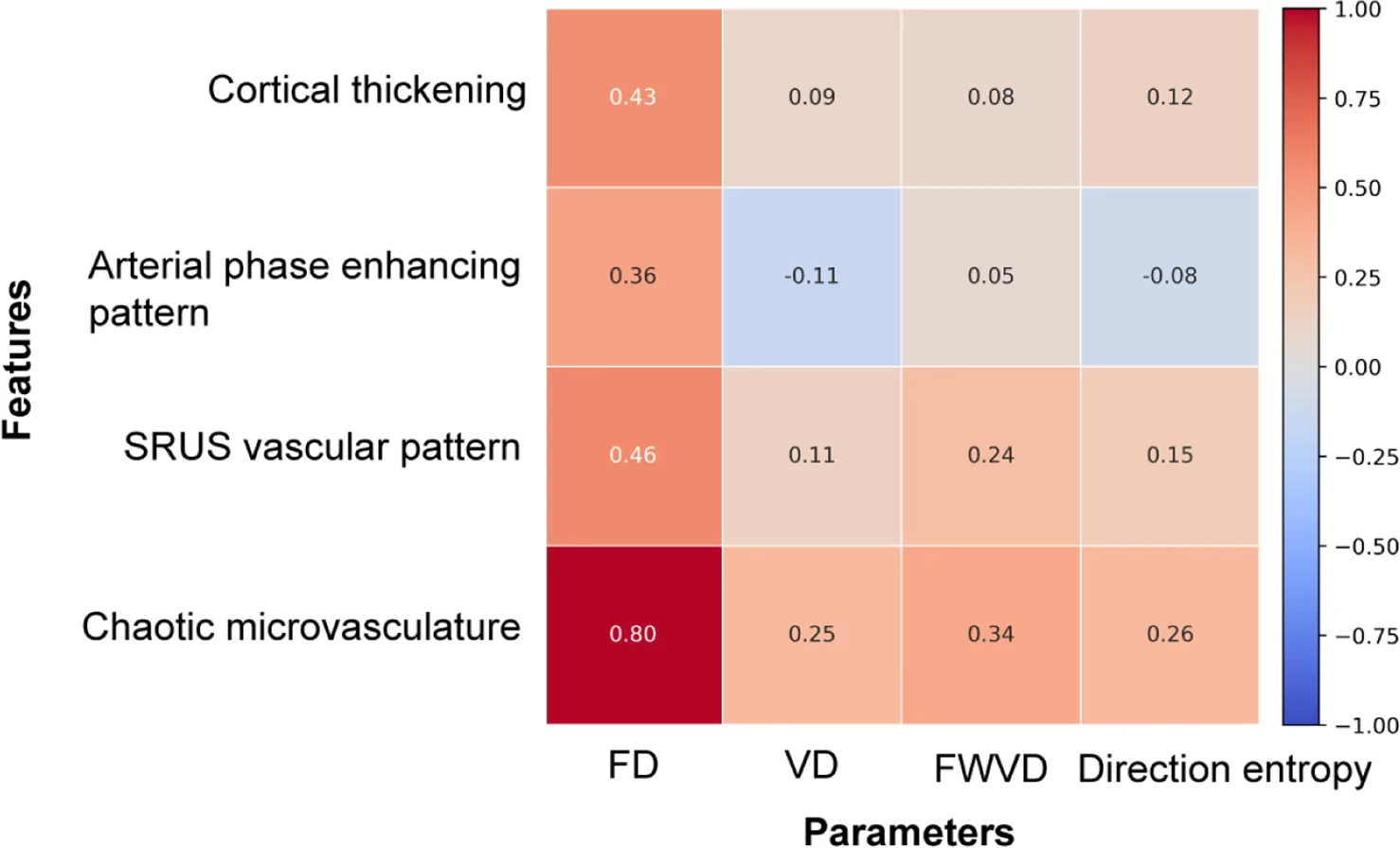
Spearman correlation analysis for major features and relevant parameters. The ρ coefficients were categorized using the following criteria: 0.00–0.09, negligible; 0.10–0.39, weak; 0.40–0.69, moderate; 0.70–0.89, strong; and 0.90–1.00, very strong correlation. FD = fractal dimension, FWVD = flow-weighted vessel density, SRUS = superresolution US, VD = vessel density.

### Correlation of Major Features and Relevant Parameters with MVD

As shown in Figure S2, CD31 staining revealed lower MVD in hyperplastic LNs than in metastatic LNs (12.48 vs 18.39; *P* < .0001). Correlation coefficients of the four major qualitative features and four relevant SRUS quantitative parameters with MVD are shown in Table S3. Among qualitative features, a strong positive correlation was observed between chaotic microvasculature and MVD (Spearman ρ = 0.82; *P* < .001). Among quantitative parameters, FD and MVD showed a moderate positive correlation (Spearman ρ = 0.65; *P* < .001). Figure 5 shows images of a metastatic LN; see Figure S3 for images of an additional metastatic LNs and Figure S4 for a hyperplastic LN.

**Figure 5:**
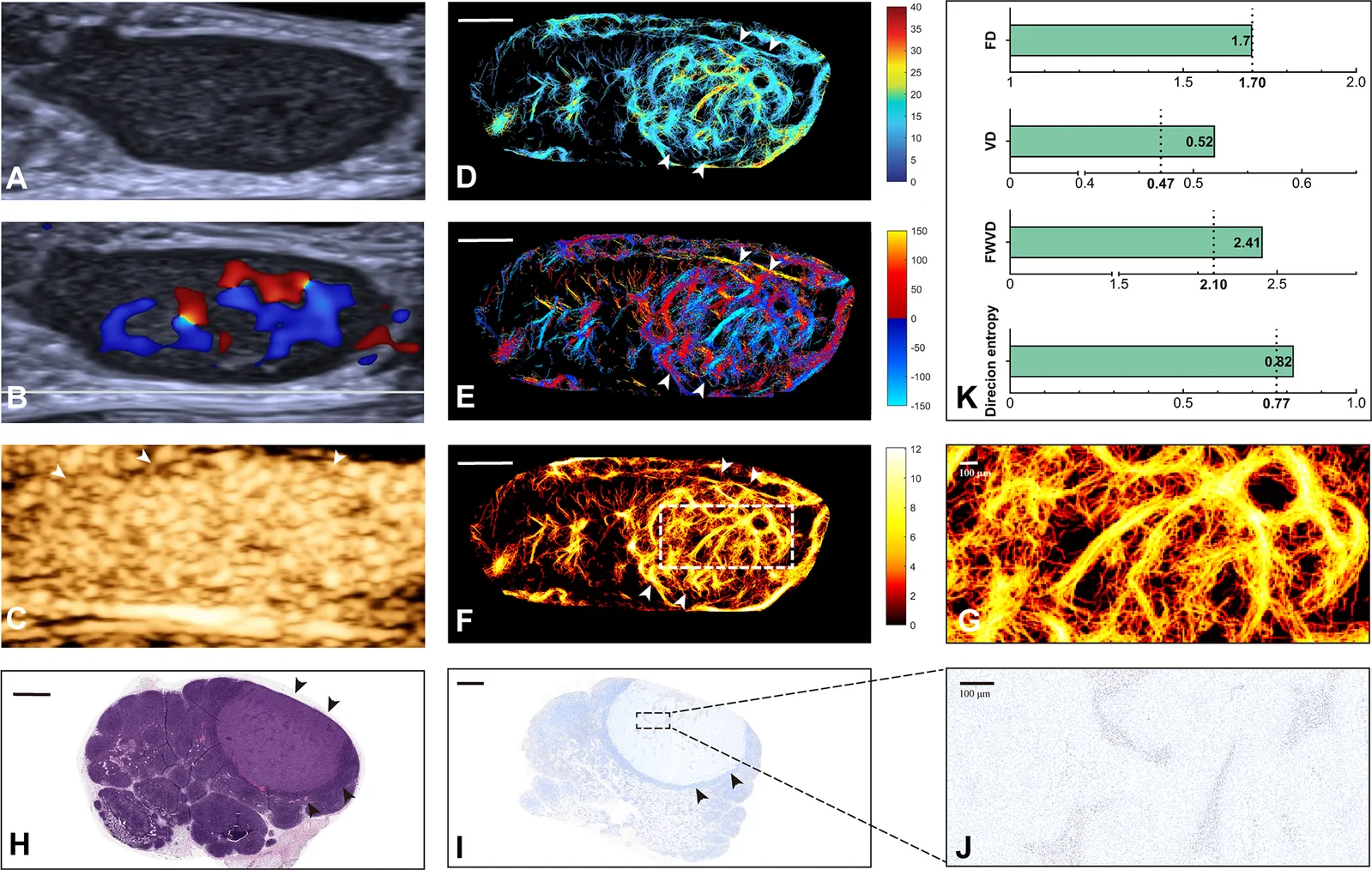
Images of a focal metastatic lymph node (LN) confirmed with histopathologic evaluation. **(A)** Grayscale image shows a 12.1 × 5.0-mm palpable LN for a rabbit. **(B)** Color Doppler flow image of the LN shows abundant blood flow and a mixed vascular pattern. **(C)** Contrast-enhanced US image of the LN shows homogeneous enhancement (white arrowheads) in the vascular phase (timer, 12 seconds). **(D–F)** Superresolution US (SRUS) velocity **(D)**, direction **(E)**, and density **(F)** maps of the LN show the mixed vascular pattern and the focal chaotic microvasculature (white arrowheads), with a higher-magnification view **(G)** of the white boxed area in panel F showing the focal chaotic microvasculature of the LN. **(H)** Hematoxylin-eosin–stained section of the LN shows a focal metastatic deposit (black arrowheads) corresponding with the focal chaotic microvasculature (white arrowheads) in SRUS maps. CD31 immunohistochemistry highlights endothelial cells (brown) outlining vascular structures within the metastatic deposit (black arrowheads), with a higher-magnification view of the boxed area in panel I shown in panel J, demonstrating dense CD31-positive microvessels corresponding to the chaotic vascular microvasculature observed in SRUS maps. **(K)** Relevant SRUS parameters of the LN. Cutoff values of each parameter are presented (dotted lines). FD = fractal dimension, FWVD = flow-weighted vessel density, VD = vessel density. Scale bars = 1 mm.

## Discussion

In this in vivo imaging study of 30 rabbits with palpable LNs, qualitative and quantitative analyses of SRUS showed that chaotic microvasculature (AUC, 0.92) and FD (AUC, 0.92) were the highest-performing qualitative feature and quantitative parameter, respectively, for the diagnosis of metastatic LNs. Chaotic microvasculature and FD were strongly correlated (Spearman ρ = 0.80; *P* < .001), with both reflecting vessel tortuosity. CD31 immunostaining also reflected the relationship between SRUS and MVD pathology. These findings suggested that SRUS-based qualitative and quantitative analyses may provide a noninvasive method of evaluating LN microvasculature to identify features associated with metastatic LNs in rabbits.

We explored the utility of SRUS in characterizing the metastatic status of LNs. SRUS enables in vivo micron-scale high-resolution imaging of microvasculature through the location and tracking of intravascular microbubbles (16,19–21). Tumors promote LN angiogenesis through increased vascular endothelial growth factor secretion and the remodeling of high endothelial venules into capillary-type vessels (16). Abnormal microvasculature, including loss of hierarchy, a chaotic network of tortuous microvessels, and high vascular density, could be observed during this angiogenetic process (10,45). This explains the underlying mechanism by which SRUS emerges as a potential imaging modality for LN metastasis and provides the theoretical foundation of our study.

In our study, qualitative analysis showed that chaotic microvasculature visually assessed at SRUS achieved the highest AUC (0.92) and moderate interreader agreement (κ = 0.60) for the diagnosis of metastatic LNs in rabbits when compared with those of conventional imaging features at US and CEUS. These results suggest that SRUS-derived qualitative features may facilitate translation of this technology into clinical practice. It could be anticipated that high-resolution SRUS adds to a radiologist’s diagnostic toolbox, enabling more accurate and reliable identification of LN metastasis through the evaluation of LN microvasculature. Given the technique’s complexity, SRUS may help characterize challenging cases or guide biopsy of LNs with focal intranodal neoplastic invasion to reduce the false-negative rate.

Regarding the results of SRUS-based quantitative analysis, FD yielded the optimal AUC (0.92) for the diagnosis of metastatic LNs, followed by VD (0.70), FWVD (0.70), and direction entropy (0.71), indicating increased vessel density, perfusion, and tortuosity. These results are generally consistent with those of a previous study (23) that suggested that the metastatic LN microvasculature has higher MVD and flow direction variance. However, results derived from algorithm-dependent quantitative analysis raise concerns regarding cross-platform reproducibility. The establishment of a unified multicenter international consensus protocol may resolve this issue, but continuous efforts are still required.

Several previous studies have explored the feasibility of applying SRUS to evaluate LN metastasis in humans (23,24). A major difference between these previous studies and our study was the manner in which SRUS and histopathologic data were correlated. In our study, we performed US-guided resection of imaged LNs to obtain whole-mount sections and aligned the tissue cutting plane with the imaging plane using ink marking, thereby ensuring spatial correlation between SRUS and histopathologic data. The other major difference was that we performed SRUS-based qualitative analyses and MVD analysis in our study. To our knowledge, this is the first attempt at the visual interpretation of SRUS microvasculature maps, the results of which were consistent with those of the quantitative analysis (Spearman ρ = 0.80; *P* < .001) and MVD (Spearman ρ = 0.82; *P* < .001).

Our study had several limitations. First, the sample size was limited to 30 rabbits, resulting in relatively wide CIs for the point estimates examined. To mitigate this limitation and increase the robustness of our statistical estimates, we used bootstrap resampling techniques. Second, VX2 tumor–bearing metastatic LNs and egg yolk–induced hyperplastic LNs in rabbits may not fully reflect the actual pathologic features of human metastatic and hyperplastic LNs. Therefore, the findings of our study cannot be directly applied to clinical settings. A small-scale human validation study is ongoing. Third, the design of our study limits the assessment of vascular changes at different metastatic stages. Future longitudinal studies are warranted to characterize microvascular dynamics during metastasis. Finally, clinical application of SRUS is further limited by its high susceptibility to physiologic motion artifacts, coupled with its strict reliance on contrast agents and computationally intensive postprocessing procedures.

In conclusion, our study demonstrated that SRUS via qualitative and quantitative analyses enabled noninvasive evaluation of LN microvasculature in rabbits, demonstrating its potential as an imaging modality to identify metastatic LNs. However, further studies are needed to validate the diagnostic performance of SRUS for differentiating metastatic from benign hyperplastic LNs in humans.

## Supplemental Files

Appendices S1-S4, Tables S1-S3, Figures S1-S4

Conflicts of Interest
