# Supplementary material for: Superresolution US Imaging of Rabbit Lymph Node Microvasculature for Metastasis Diagnosis
Source: Radiol Imaging Cancer. 2026 Sep 25;8(5):e250674. doi: 10.1148/rycan.250674 (PMC13620326; doi:10.1148/rycan.250674)
Supplement: Supplementary file 1 [file rycan250674suppa1.pdf]

Supplemental materials undergo peer review but are not copyedited, and may include typographical errors.

©RSNA, 2026
10.1148/rycan.250674

Appendix S1

The Reason for VX2 Tumor Selection in LN Model Establishment

The rabbit VX2 tumor, a well-characterized squamous cell carcinoma, has been extensively utilized in oncology research in lung (46), liver (47), lymph nodes (33,48,49). Despite species distinction from human malignancies, VX2 tumors share the way to metastasize mainly by lymphatic way (50). Furthermore, the macroscopic sizes and regional distribution of rabbit LNs are highly comparable to those in humans, making it an ideal preclinical model for simulating clinical diagnostic scenarios.

The Detailed Procedure of Metastatic LN Modeling

VX2 tumor (JNO-Q0030-1+, Jennio) was previously cultured in the right thigh muscle of a donor rabbit. After four weeks, the donor rabbit was euthanized by an intravenous anesthetic overdose. The active tumor tissue was then excised, minced into approximately 1.0-mm^3 pieces, and suspended in normal saline (10% w/v).

For modeling, recipient rabbits were initially anesthetized with an intramuscular injection of 0.1 mL/kg xylazine hydrochloride solution (Sumianxin II; Sheng Xin) for induction. Anesthesia was then maintained with an intravenous injection of zolazepam/tiletamine hydrochloride solution (Zoletil®50; Virbac) at a dose of 0.1 mL/(kg•h). The rabbits were placed supine on a heated platform. After hair removal and disinfection, 1.0 mL of the tumor suspension was subcutaneously injected into the right 2nd mammary gland using an 18-G needle. The injection site was finally disinfected again.

The Detailed Procedure of Hyperplastic LN Modeling

Subcutaneous injection of a 10% egg-yolk emulsion is a standard method for inducing benign LN

Supplemental materials undergo peer review but are not copyedited, and may include typographical errors.

hyperplasia. The emulsion was prepared by mixing fresh egg yolk with normal saline (10% v/v) with complete oscillation.

Previous protocols injected 0.5 mL of the emulsion into the submandibular region to target cervical nodes (29,30). To target the axillary lymphatic drainage basin instead, we modified this protocol by subcutaneously injecting 1.0 mL of the emulsion into the right 2nd mammary gland. All other perioperative procedures, including the intravenous anesthesia, were identical to the metastatic LN modeling protocol.

Appendix S2

Protocols for US, CDFI, CEUS and SRUS Examinations

Ultrasound examinations were performed using a clinical scanner (Resona A20, Mindray) equipped with an LM18-5WU linear array transducer (5-18 MHz) and an SL10-3U linear array transducer (3-10 MHz). First, conventional B-mode US and color Doppler flow imaging (CDFI) examinations with the high-frequency LM18-5WU transducer were initially performed for localizing the target palpable lymph node (LN), determining its maximal cross-section and assess the baseline blood flow. Next, the SL10-3U linear array transducer was selected for the subsequent CEUS and SRUS imaging. This transducer was specifically chosen because its frequency bandwidth better matches the resonance frequency of perfluorobutane microbubbles (Sonazoid, GE Healthcare) arranging 4-4.5 MHz, thereby optimizing the generation of non-linear contrast signals (35). The image depth and gain were optimized, a contrast-specific imaging mode with a low mechanical index (MI) was strictly applied to extract these non-linear signals while minimizing microbubble destruction.

For CEUS and SRUS image acquisition, the perfluorobutane microbubbles (Sonazoid, GE

Supplemental materials undergo peer review but are not copyedited, and may include typographical errors.

Healthcare) were prepared according to the manufacturer's instructions. A rapid bolus injection of perfluorobutane microbubbles (0.04 mL/kg) was injected into the marginal ear vein, followed immediately by a 3-mL saline flush. A timer was started immediately after the injection. The transducer was kept completely stable at the predefined maximal cross-section of the LN. A continuous CEUS cine loop was acquired for the initial approximately 60 seconds (frame rate, 12 Hz; MI, 0.129).

When the target LN microvasculature was adequately perfused and imaged on CEUS (after the initial 60 seconds), the SRUS protocol was immediately initiated. The transducer was kept strictly stationary to minimize motion artifacts. SRUS continuous raw data were acquired for 5 seconds at an ultra-high frame rate of 500 Hz, with a lower MI of 0.113 used to preserve the contrast agent during the high-frame-rate acquisition.

SRUS Postprocessing

Raw data was input in SRUS Platform software (version 3.0, Mindray), during which the motion estimation and compensation were carried out. Prior to reconstruction, the processing frame range was manually set from frame 0 to 2500, and the sequence was previewed via playback to ensure the stability of the acquired imaging plane. Furthermore, the "V2" imaging mode was selected for processing, as it provides a higher imaging resolution compared to the baseline V1 mode. Then SRCEUS Platform started to reconstruct the vasculature using 2500 compounded B-mode images by detecting and tracking microbubbles (MBs) over several frames precisely. The tracking procedure yielded the trajectories of MBs, which were accumulated to generate the SRUS density map, velocity map and direction map.

Supplemental materials undergo peer review but are not copyedited, and may include typographical errors.

Appendix S3

The Definitions of SRUS Quantitative Parameters

Fractal Dimension

Fractal dimension (FD) is a mathematical metric used to quantify the complexity and self-similarity of fractal structures based on box-counting method (51). FD is used to describe the complexity of an image, which is quantified as the ratio of the changes in graphic detailed to the changes in measurement scale. The higher the value of FD is, the higher the complexity of image is. FD in the region of interest (ROI) is calculated based on the SRUS density map, reflecting the complexity of blood vascular morphology in the ROI.

$$FD = \lim_{S \to 0} \frac{log\, N(s)}{log(\frac{1}{s})}$$

Where N is the number of self-similarities at a given scale, and s is the scaling factor associated with the size of the object at different scales.

Vessel Density

Vessel Density (VD) is defined as the proportion of vascular pixels relative in the binarized image to total pixels within a manually selected ROI.

$$VD = \frac{vessel\ pixels}{overall\ pixels}$$

Where $vessel\ pixels =$ Number of vascular pixels, $overall\ pixels =$ Total pixel counts in ROI.

Supplemental materials undergo peer review but are not copyedited, and may include typographical errors.

Flow Weighted Density of The Vessels

Flow Weighted Density of the vessels (FWVD) is defined as the ratio of the sum of the pixel values (density) of blood vessels to the total number of pixels in the ROI. FWVD represents perfused blood volume, with higher values indicating higher blood flow volume.

$$FWVD = \frac{\sum pixel\ values}{overall\ pixels}$$

Direction Entropy

Entropy can reflect the degree of chaos in the ROI; the greater the entropy is, then the more information the image contains and more complex the content is. Local flow direction entropy is quantified on blood flow direction map.

$$Direction\ Entropy = -\sum_{i}^{N} p(d_i) \log p(d_i)$$

Where N is the number of tracking MBs, d_i represents the direction of the ith MB, and $p(d_i)$ is the probability of d_i occurrence in ROI.

Velocity

Blood flow velocity was quantified by the vector mean of passed MB-trajectories. Mean velocity was taken as the average value of the sum flow velocity calculated from every tracking MB, while peak velocity is the highest velocity. Velocity variance was obtained by calculating the variance of MBs' velocities which could reflect the degree of velocity distribution in the ROI.

$$Velocity\ variance = \frac{1}{N-1}\sum_{i=1}^{N} |v_i - \bar{v}|^2$$

Supplemental materials undergo peer review but are not copyedited, and may include typographical errors.

Where N is the number of tracking MB, v_i represents the velocity of the ith MB, and $\bar{v}$ represents the mean velocity.

Perfusion Index

Perfusion index is defined as the product of the mean blood flow velocity in the ROI multiplied by blood vessel density which reflects the perfusion level.

$$PI = \bar{v} \times VD$$

Where $\bar{v}$ represents the mean velocity.

Appendix S4

Statistical Analysis Plan

Sample size calculation was performed by using software (PASS 2023; NCSS). The area under the receiver operating characteristic curve (AUC) of the SRUS for identifying metastatic LN was the primary outcome measure. A sample of 14 from the metastasis LN group and 14 from the hyperplasia LN group produce a 95% CI with a width of 0.30 when the expected AUC is 0.85. Allowing for a 10% dropout rate, at least 32 rabbits (16 per group) are required.

Categorical variables are presented as frequencies and percentages, and compared between groups using the χ^2 test or Fisher exact test. Continuous variables are reported as either mean ± standard deviation (SD) or median with interquartile ranges (IQRs) in parentheses as appropriate, and compared between groups using the Student t test or Mann-Whitney U test. Interreader agreement for individual qualitative features was assessed using Cohen's κ statistics with 95% confidence intervals (CIs). Intrareader reproducibility of three measurements of quantitative

Supplemental materials undergo peer review but are not copyedited, and may include typographical errors.

parameters was evaluated using intraclass correlation coefficients (ICCs) with 95% CIs. *κ* coefficients were categorized according to Landis and Koch using following criteria (42): 0.00-0.20, slight agreement; 0.21-0.40, fair agreement; 0.41-0.60, moderate agreement; 0.61-0.80, good agreement; and 0.81-1.00, excellent agreement. ICCs were interpreted according to Koo and Li as follows (43): less than 0.50, poor; 0.51-0.75, moderate; 0.76-0.90, good; and greater than 0.90, excellent. The major qualitative features and relevant SRUS quantitative parameters were identified by univariable binary logistic regression analysis, with the odds ratios (ORs) corrected using Firth's method and internally validated using 1000 bootstrap samples. Qualitative features or quantitative parameters with significantly associated with metastatic risk ($P < .05$) were considered major features or relevant parameters. The diagnostic performance was evaluated separately: the Dorfman-Berbaum-Metz (DBM) method was used for qualitative features interpreted by multiple readers, whereas standard ROC analysis was applied to the quantitative parameters measured by a single radiologist, with AUC serving as the overall performance metric (52,53). Optimal cutoff values for the quantitative parameters were determined by maximizing Youden's index. The 95% CIs for sensitivity, specificity, positive predictive value, negative predictive value, and AUCs were computed and compared using bootstrap method with 1000 resamples. For extreme diagnostic proportions (i.e., 100% accuracy) where standard bootstrap yields zero-variance intervals, the Wilson score interval method was applied. The relationships between major qualitative features, relevant SRUS quantitative parameters, and histopathological microvascular density (MVD) were evaluated using the Spearman rank correlation coefficient (*r*) according to Schober as follows (44): 0.00-0.09, negligible; 0.10-0.39, weak; 0.40-0.69, moderate; 0.70-0.89, strong; and 0.90-1.00, very strong correlation. *P* values were adjusted with false discovery rate (FDR) method for multiple

Supplemental materials undergo peer review but are not copyedited, and may include typographical errors.

comparisons. A two-tailed P value of < .05 was considered statistically significant.

All statistical analyses were performed using SPSS software (version 31.0; IBM), and Python (version 3.12.7; Python Software Foundation).

Supplemental materials undergo peer review but are not copyedited, and may include typographical errors.

References

46. Ueki A, Okuma T, Hamamoto S, Miki Y. Therapeutic Effects of CT-guided Radiofrequency Ablation with Concurrent Platinum-Doublet Chemotherapy in a Rabbit VX2 Lung Tumor Model. Radiology. 2017;283(2):391–398. doi: 10.1148/radiol.2016160414.

47. Van Breugel JMM, Geschwind J-F, Mirpour S, et al. Theranostic application of lipiodol for transarterial chemoembolization in a VX2 rabbit liver tumor model. Theranostics. 2019;9(13):3674–3686. doi: 10.7150/thno.32943.

48. Niu C, Wang Z, Lu G, et al. Doxorubicin loaded superparamagnetic PLGA-iron oxide multifunctional microbubbles for dual-mode US/MR imaging and therapy of metastasis in lymph nodes. Biomaterials. Elsevier; 2013;34(9):2307–2317.

49. Choi SH, Han MH, Moon WK, et al. Cervical Lymph Node Metastases: MR Imaging of Gadofluorine M and Monocrystalline Iron Oxide Nanoparticle–47 in a Rabbit Model of Head and Neck Cancer. Radiology. 2006;241(3):753–762. doi: 10.1148/radiol.2413051979.

50. Kidd JG, Rous P. A transplantable rabbit carcinoma originating in a virus-induced papilloma and containing the virus in masked or altered form. J Exp Med. 1940;71(6):813–838. doi: 10.1084/jem.71.6.813.

51. Hayano K, Lee SH, Yoshida H, Zhu AX, Sahani DV. Fractal Analysis of CT Perfusion Images for Evaluation of Antiangiogenic Treatment and Survival in Hepatocellular Carcinoma. Academic Radiology. 2014;21(5):654–660. doi: 10.1016/j.acra.2014.01.020.

52. Hillis SL. A comparison of denominator degrees of freedom methods for multiple observer ROC analysis. Stat Med. 2007;26(3):596–619. doi: 10.1002/sim.2532.

53. Hillis SL, Berbaum KS, Metz CE. Recent developments in the Dorfman-Berbaum-Metz procedure for multireader ROC study analysis. Acad Radiol. 2008;15(5):647–661. doi: 10.1016/j.acra.2007.12.015.

Supplemental materials undergo peer review but are not copyedited, and may include typographical errors.

Table S1. Interreader agreement for US, CEUS and SRUS features

Features	κ value	Agreement level
**US**		
Shape	0.38 (0.00, 0.78)	Fair
Echogenicity	0.46 (0.10, 0.77)	Moderate
Lymphatic hilum	0.29 (0.04, 0.60)	Fair
Cortical thickening	0.40 (0.04, 0.71)	Fair
CDFI vascular pattern	0.50 (0.25, 0.71)	Moderate
**CEUS**		
Perfusion pattern	0.38 (0.18, 0.58)	Fair
Arterial phase enhancing pattern	0.55 (0.21, 0.84)	Moderate
Perfusion defect	0.67 (0.22, 1.00)	Good
**SRUS**		
SRUS vascular pattern	0.51 (0.15, 0.83)	Moderate
Chaotic microvasculature	0.60 (0.27, 0.87)	Moderate

Note: data in parentheses are 95% CIs.

κ coefficients were categorized using following criteria: 0.00–0.20, slight agreement; 0.21–0.40, fair agreement; 0.41–0.60, moderate agreement; 0.61–0.80, good agreement; and 0.81–1.00, excellent agreement.

Supplemental materials undergo peer review but are not copyedited, and may include typographical errors.

Table S2. Intrareader Reproducibility of quantitative parameters.

Quantitative Parameters	Intraobserver ICC	Reliability
FD	0.86 (0.76, 0.93)	Good
VD	0.92 (0.85, 0.96)	Excellent
FWVD	0.92 (0.86, 0.96)	Excellent
Direction entropy	0.88 (0.80, 0.94)	Good
Mean velocity	0.94 (0.88, 0.97)	Excellent
Velocity variance	0.96 (0.93, 0.98)	Excellent
Peak velocity	0.95 (0.91, 0.97)	Excellent
PI	0.92 (0.86, 0.96)	Excellent

Note.—Data in parentheses are 95% CIs. Reproducibility based on ICC estimates was interpreted as follows: less than 0.50, poor; 0.50-0.75, moderate; 0.76-0.90, good; and greater than 0.90, excellent. CI = confidence interval, FD = fractal dimension, FWVD = flow-weighted density of the vessels, ICC = intraclass correlation coefficient, PI = perfusion index, VD = vessel density.

Supplemental materials undergo peer review but are not copyedited, and may include typographical errors.

Table S3. Correlation of major features and relevant SRUS parameters with MVD.

	Spearman correlation coefficients (r)	P value
Major Features		
Cortical thickening	0.44 (0.17, 0.68)	.02*
Arterial phase enhancing pattern	0.80 (0.67, 0.87)	<.001*
SRUS vascular pattern	0.52 (0.23, 0.77)	.003*
Chaotic microvasculature	0.82 (0.68, 0.87)	<.001*
Relevant SRUS Parameters		
FD	0.65 (0.37, 0.82)	<.001*
VD	0.22 (-0.16, 0.55)	.24
FWVD	0.27 (-0.11, 0.58)	.15
Direction entropy	0.26 (-0.12, 0.57)	.17

Note: data in parentheses are 95% CIs.

FD = fractal dimension, FWVD = flow weighted density of the vessels, MVD = microvascular density, VD = vessel density.

* Statistically significant.

Supplemental materials undergo peer review but are not copyedited, and may include typographical errors.

Supplemental figure legends

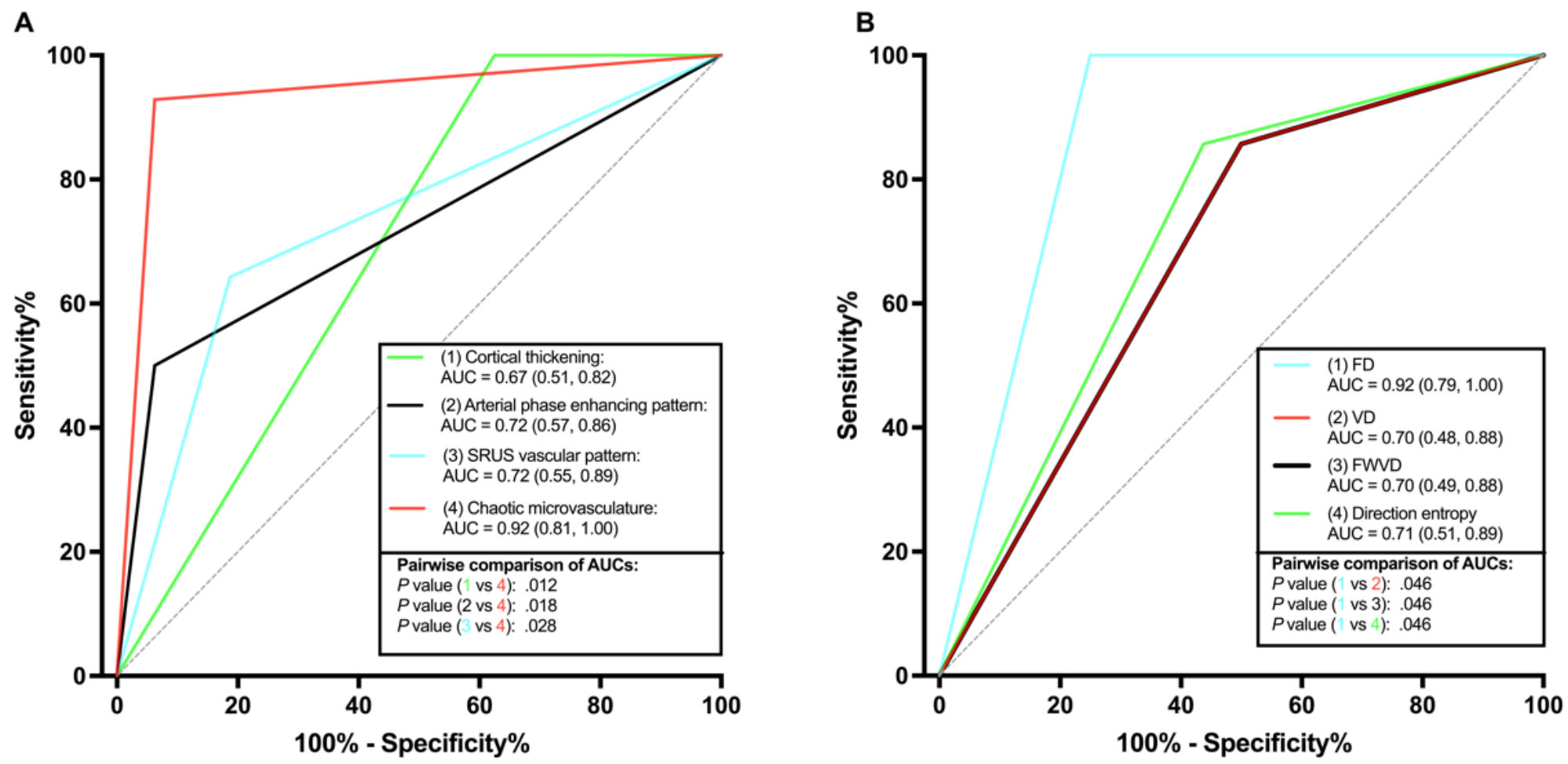

Figure S1: (A) Receiver operating characteristic curves show the diagnostic performance of major imaging features with AUCs and 95% CI in parentheses. **(B)** Receiver operating characteristic curves show the diagnostic performance of super-resolution US (SRUS) relevant parameters with AUCs and 95% CI in parentheses. FD = fractal dimension, FWVD = flow weighted density of the vessels, VD = vessel density.

Supplemental materials undergo peer review but are not copyedited, and may include typographical errors.

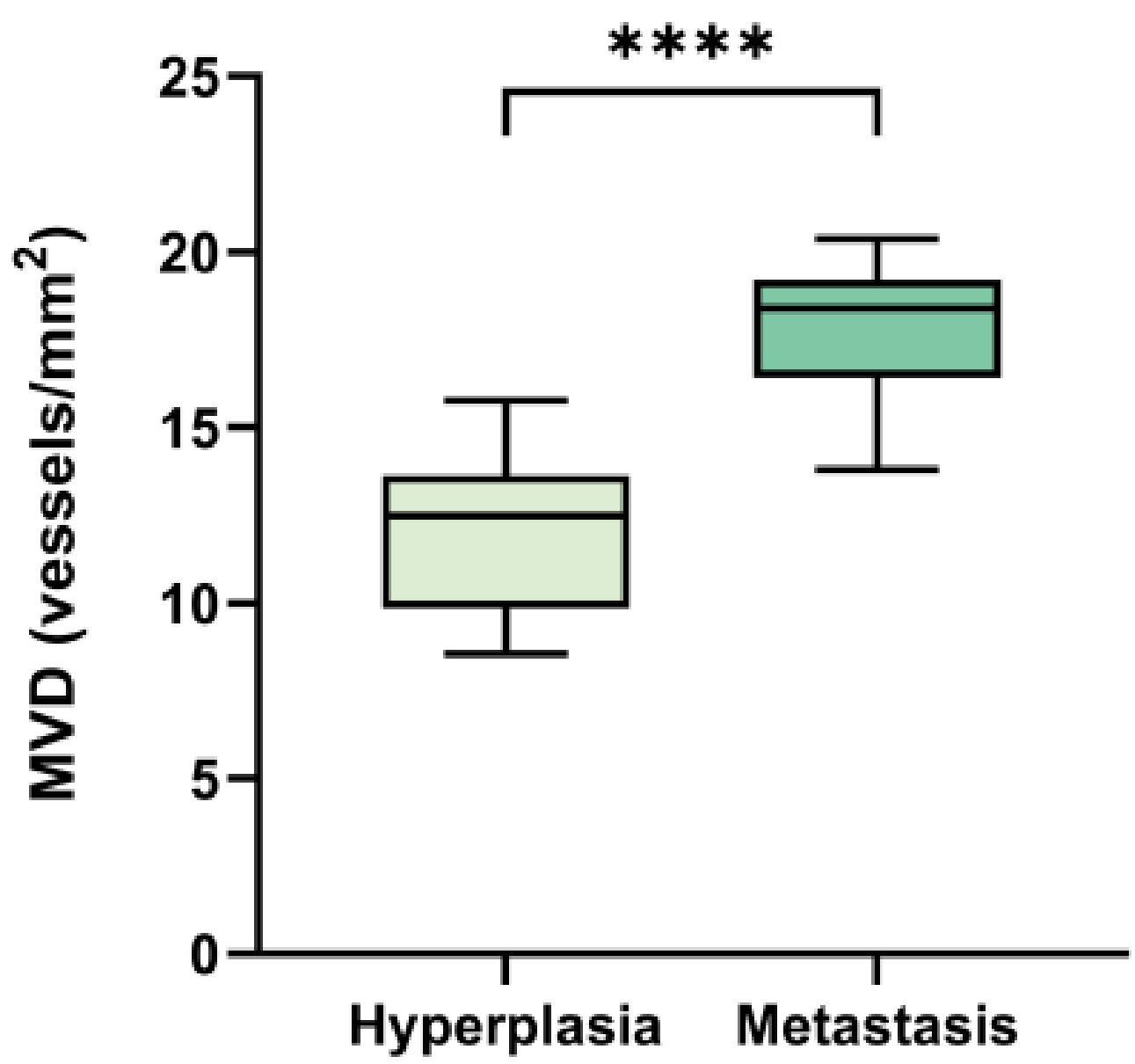

Figure S2: Box and whisker plot shows the comparison of the microvascular density. Box borders show the 25th and 75th percentiles, midlines indicate the median, and whiskers refer to the minimum and maximum values. **** P < .0001.

Supplemental materials undergo peer review but are not copyedited, and may include typographical errors.

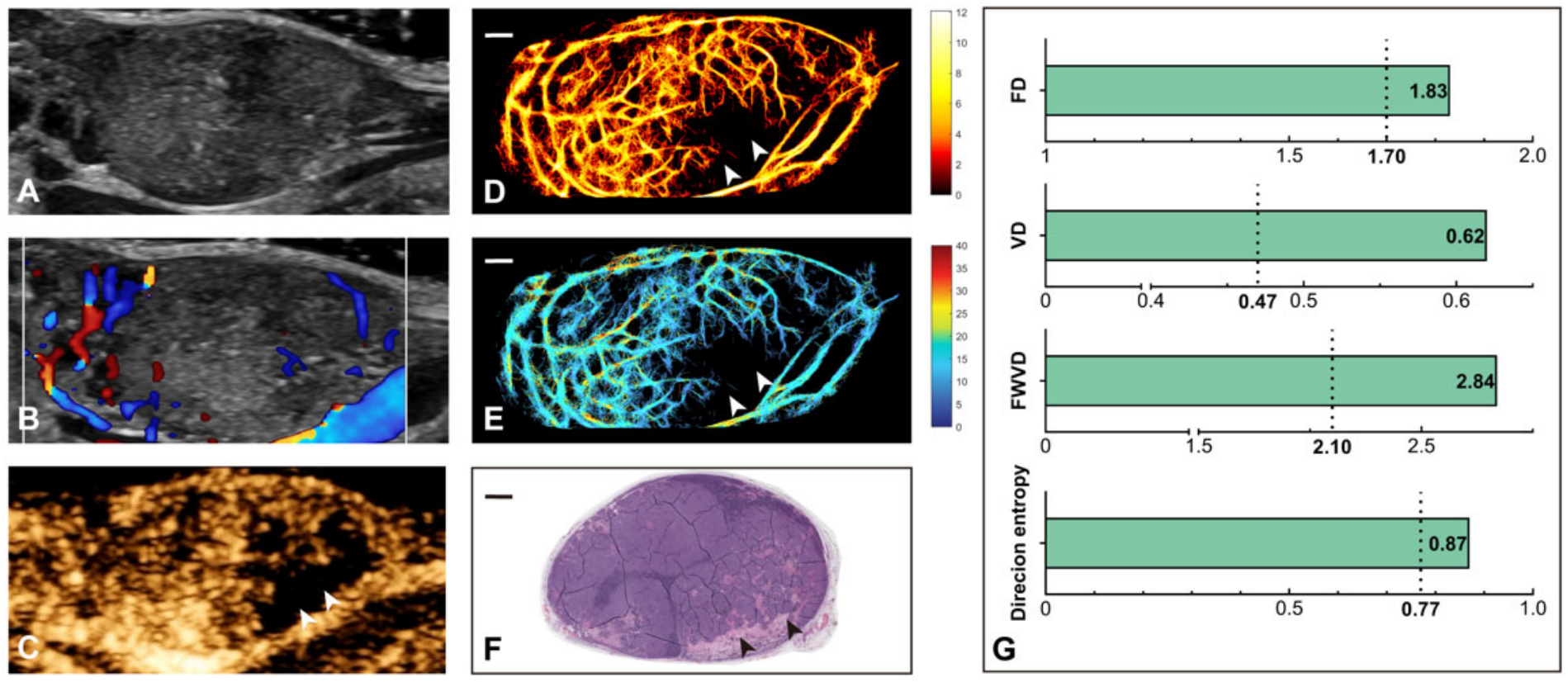

Figure S3: Images of an integral metastatic lymph node (LN) confirmed by histopathological diagnosis. **(A)** Gray-scale image shows a 17.8×8.8-mm palpable LN for rabbits. **(B)** Color Doppler flow imaging (CDFI) image of the LN shows few blood flows and peripheral vascular pattern. **(C)** Contrast-enhanced US (CEUS) image of the LN shows heterogeneous enhancement and perfusion defect (white arrowheads) in the vascular phase (timer, 7 seconds). **(D-E)** Super-resolution US (SRUS) density and velocity maps of the LN show the mixed vascular pattern and the integral chaotic microvasculature with focal vascularity defection (white arrowheads). **(F)** H&E slide of the LN shows an integral metastatic deposit with necrosis (black arrowheads) corresponding with the perfusion defect in CEUS and the focal vascularity defection in SRUS maps. **(G)** SRUS parameters of the LN. Cutoff values of each parameter are presented (dotted lines). FD = fractal dimension, FWVD = flow weighted density of the vessels, VD = vessel density. Scale bars = 1 mm.

Supplemental materials undergo peer review but are not copyedited, and may include typographical errors.

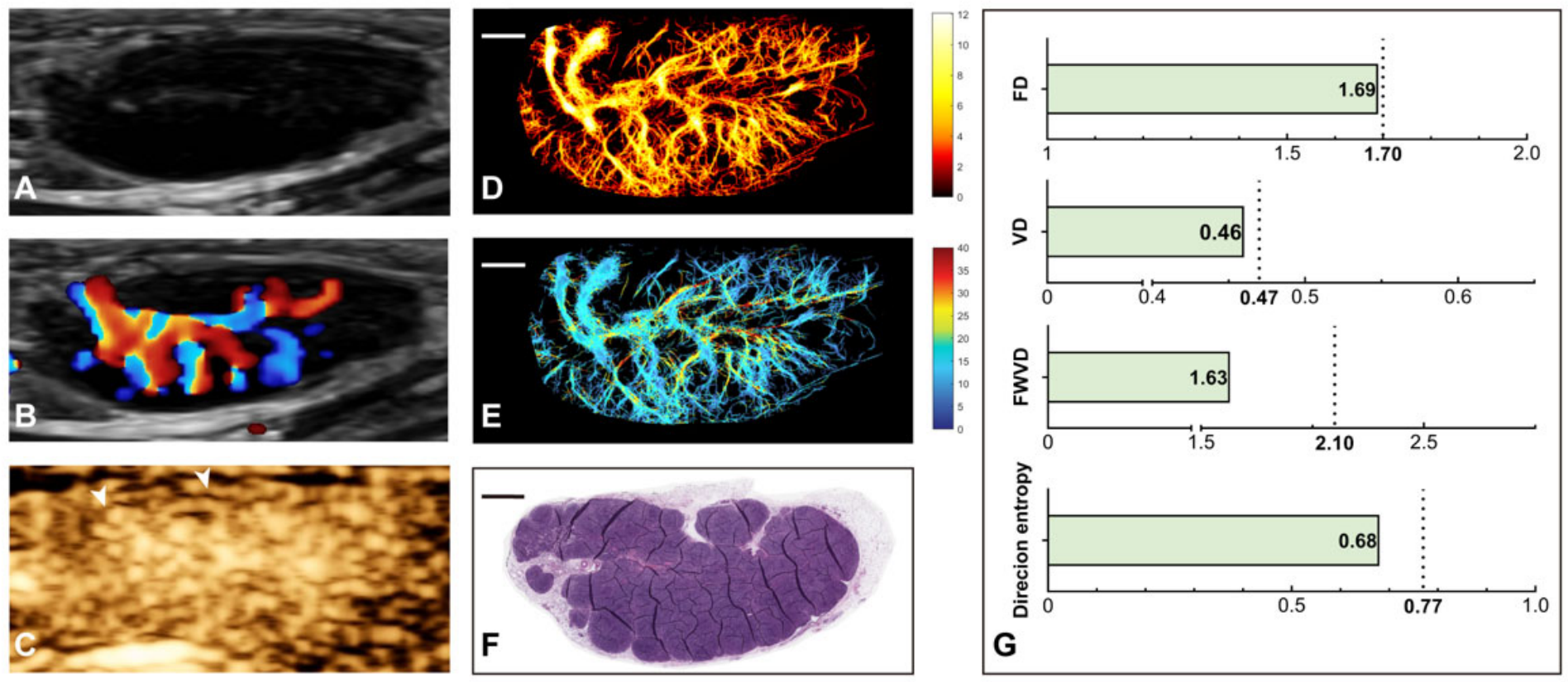

Figure S4: Images of a hyperplastic lymph node (LN) confirmed by histopathological diagnosis. **(A)** Gray-scale image shows a 9.6×3.7-mm palpable LN for rabbits. **(B)** Color Doppler flow imaging (CDFI) image of the LN shows abundant blood flows and hilar vascular pattern. **(C)** Contrast-enhanced US (CEUS) image of the LN shows homogeneous enhancement (white arrowheads) in the vascular phase (timer, 12 seconds). **(D-E)** Super-resolution US (SRUS) density and velocity maps of the LN show the hilar vascular pattern and the absence of chaotic microvasculature. **(F)** H&E slide of the LN shows no metastatic deposits. **(G)** Relevant SRUS parameters of the LN. Cutoff values of each parameter are presented (dotted lines). FD = fractal dimension, FWVD = flow weighted density of the vessels, VD = vessel density. Scale bars = 1 mm.
